# Supplementary material for: Integrative epigenomic and high-throughput functional enhancer profiling reveals determinants of enhancer heterogeneity in gastric cancer
Source: Genome Med. 2021 Oct 11;13:158. doi: 10.1186/s13073-021-00970-3 (PMC8504099; doi:10.1186/s13073-021-00970-3)
Supplement: Supplementary file 2 — Additional file 2: Supplementary Figures and Figure legends S1-S26. [file 13073_2021_970_MOESM2_ESM.pdf]

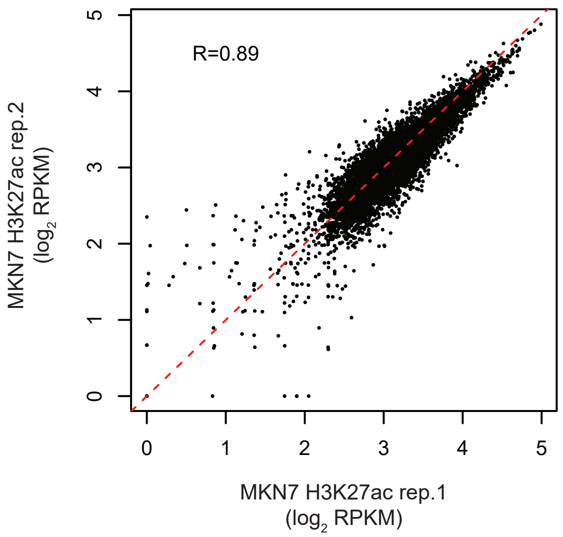

Supplementary Figure 1: Comparison of H3K27ac signals over common predicted enhancers between two MKN7 replicates.

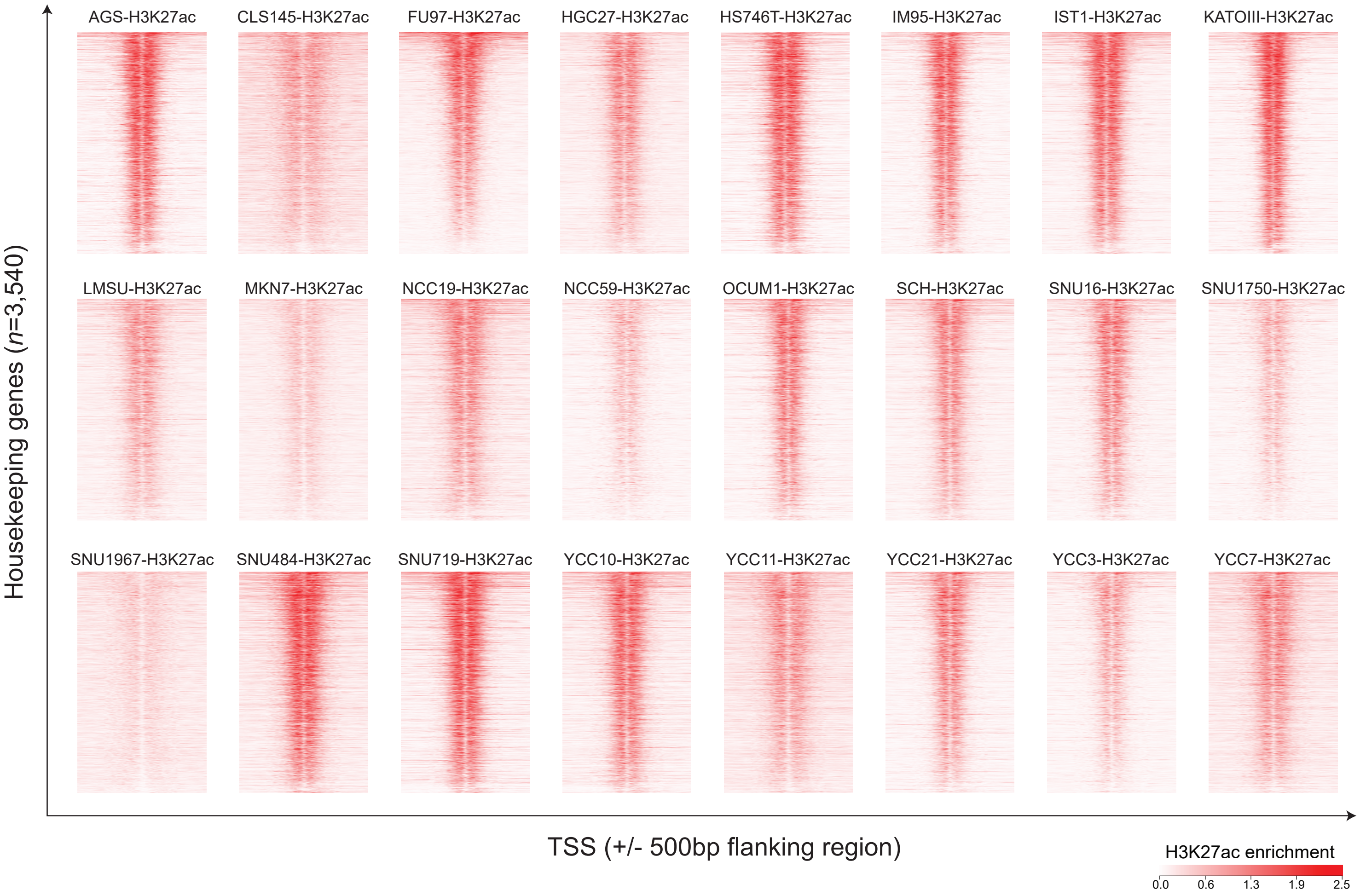

Supplementary Figure 2: H3K27ac enrichment at regions flanking TSS of housekeeping genes. All cell lines in this study demonstrated H3K27ac enrichment at regions flanking the TSS of 3,504 housekeeping genes. H3K27ac ChIP-seq was visualized using NGSplot, and the list of housekeeping genes were derived from Eisenberg & Levanon, 2013.

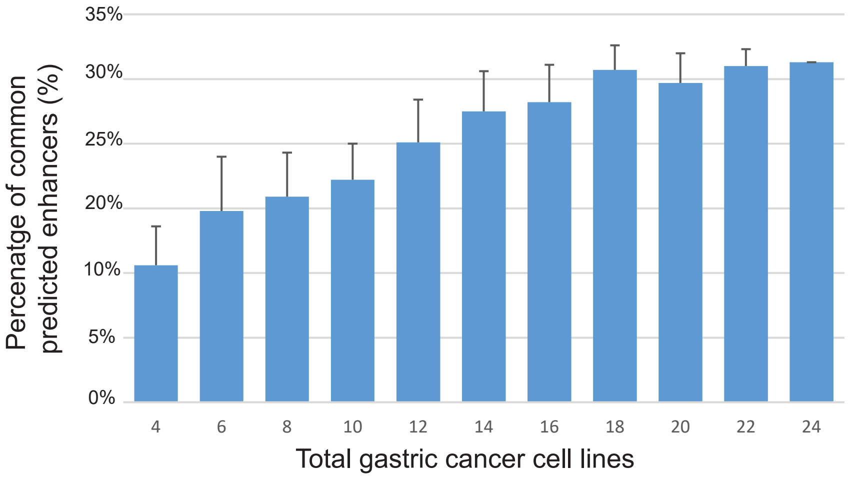

Supplementary Figure 3: Recurrence rates of predicted enhancers. Recurrent enhancers here are defined as those enhancers occuring in at least 3 GC cell lines Data presented are the mean percentage +/- standard deviation of common predicted enhancers found in three or more gastric cancer cell lines, as a function of number of cell lines.

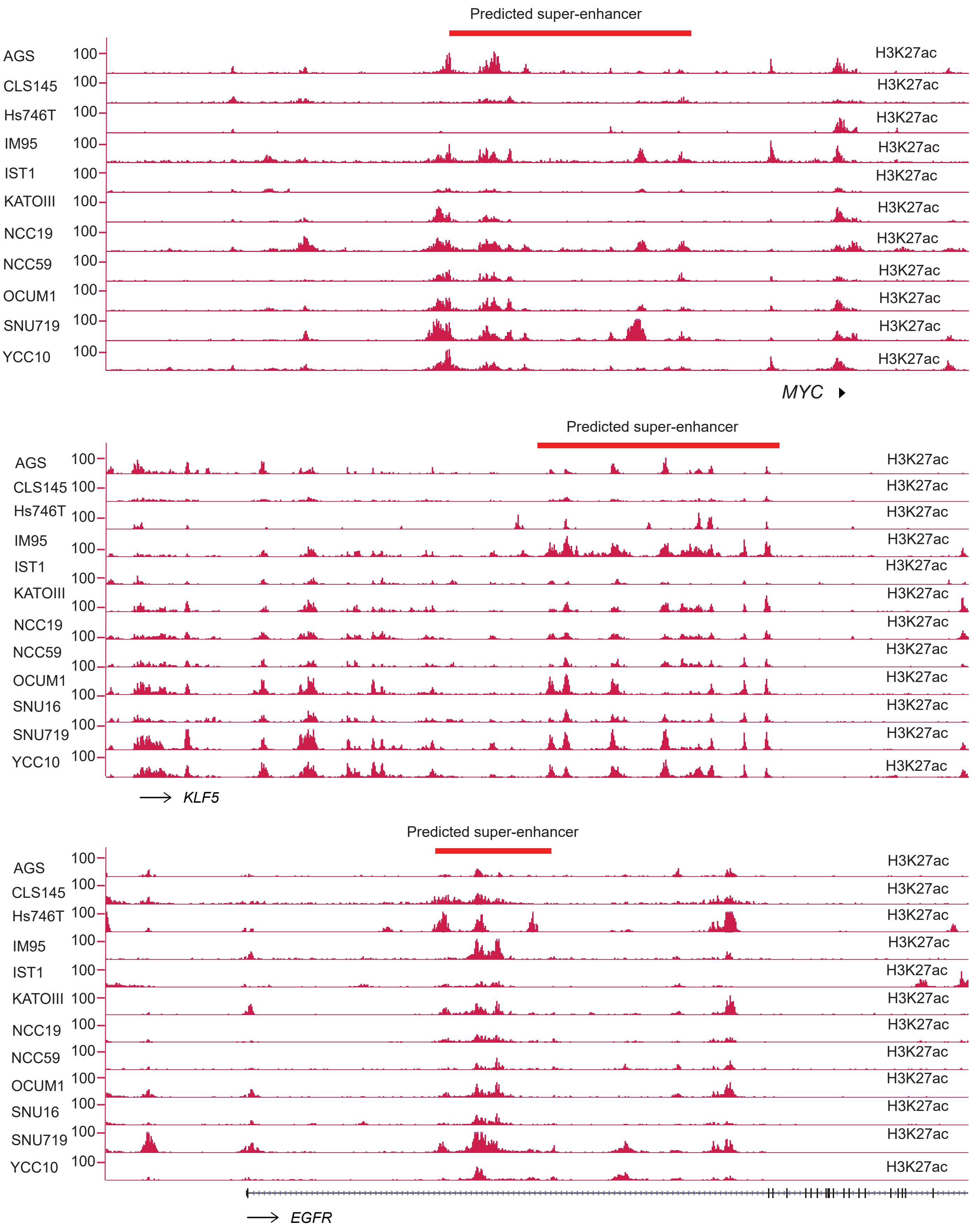

Supplementary Figure 4: Predicted super-enhancers in GC cell lines. *MYC*-, *KLF5*- and *EGFR*-associated predicted super-enhancers are recurrent in multiple GC cell lines.

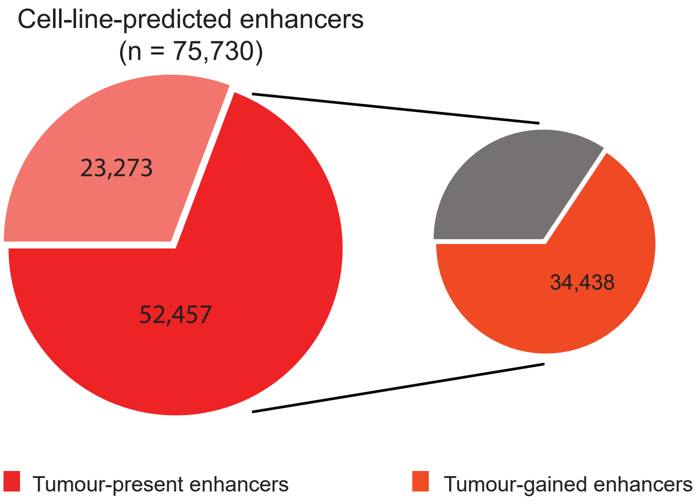

Supplementary Figure 5. Distribution of cell-line-predicted enhancers in GCs. Tumour-present enhancers represent those enhancers present in GCs. Tumour-gained enhancers represent those enhancers exhibiting somatic gain in two or more primary GCs.

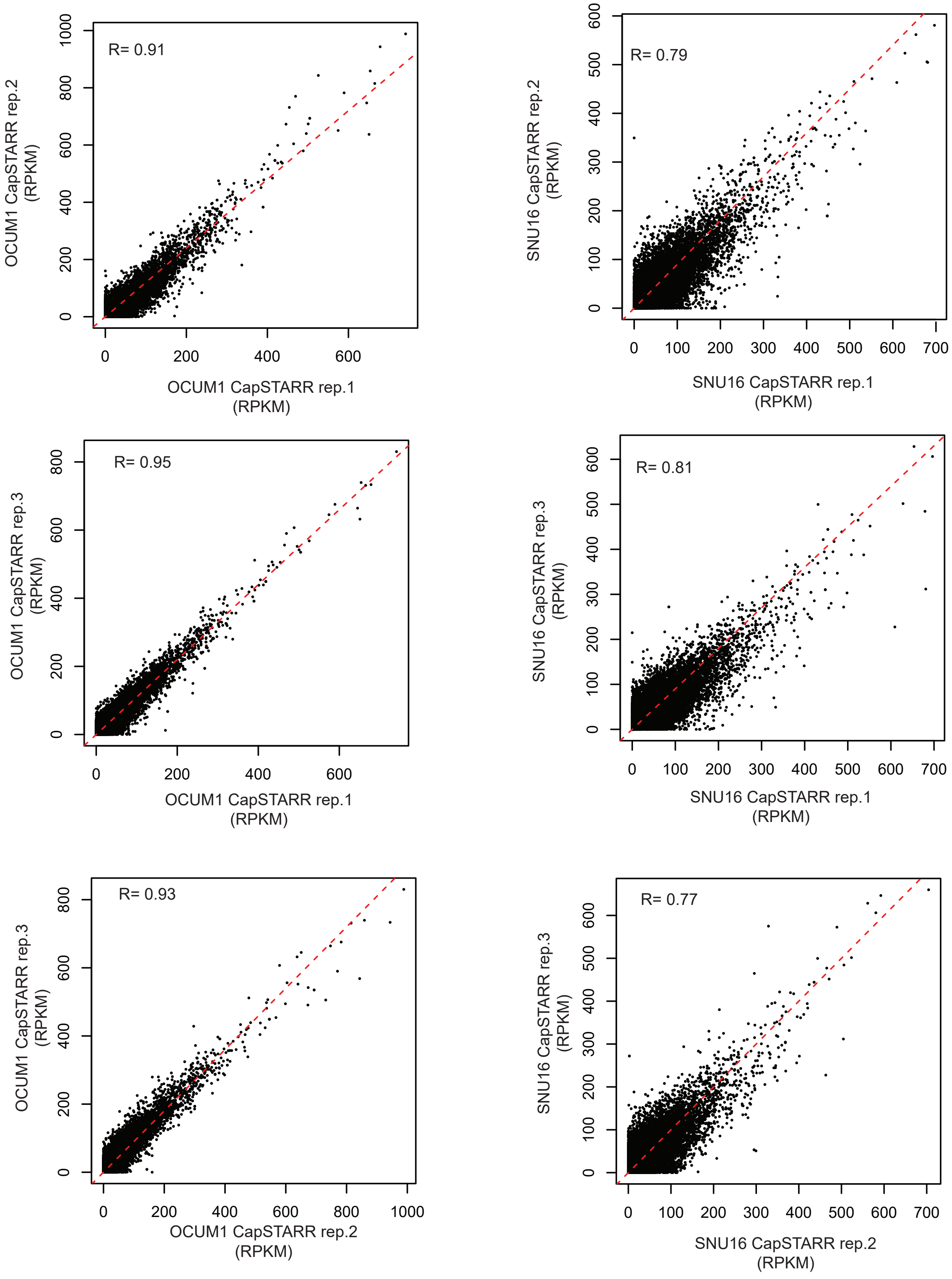

Supplementary Figure 6: Comparison of CapSTARR-seq signals over CapSTARR-seq enriched regions between OCUM1 (left panels) or SNU16 (right panels) replicates.

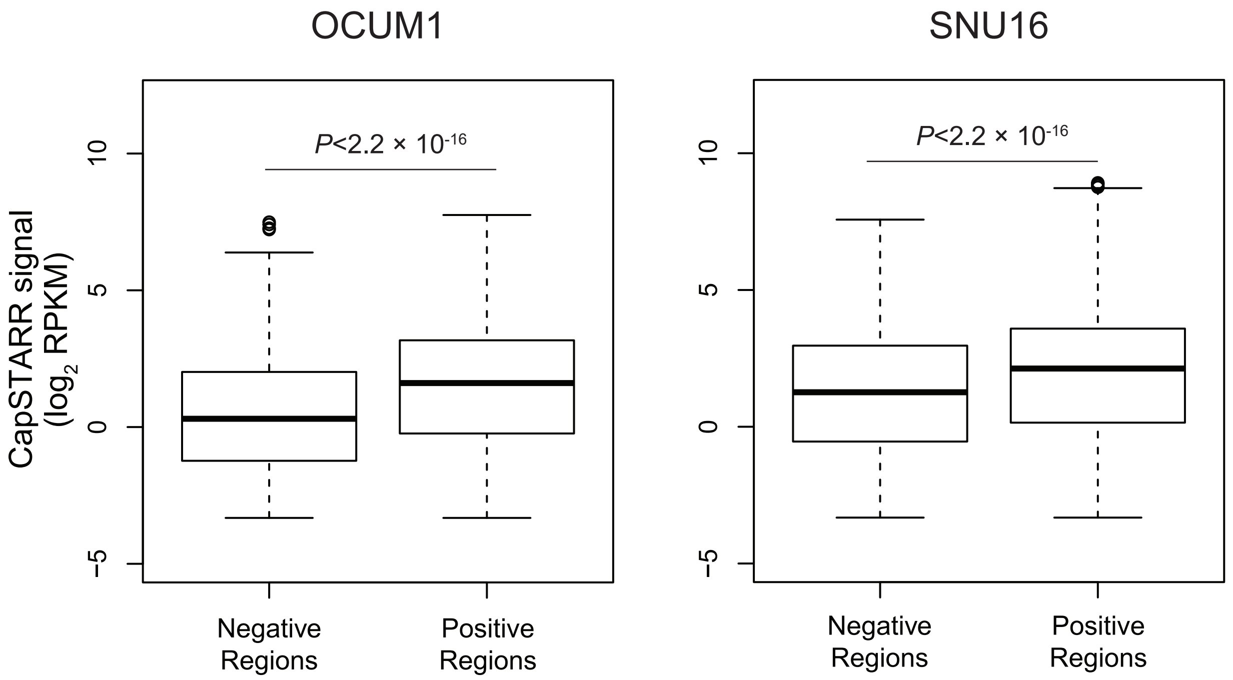

Supplementary Figure 7: Differences in corrected CapSTARR-seq signals (log_2 RPKM) over captured predictive enhancers (positive) and negative regions in OCUM1 and SNU16 cells. *P*-values: Mann–Whitney U test.

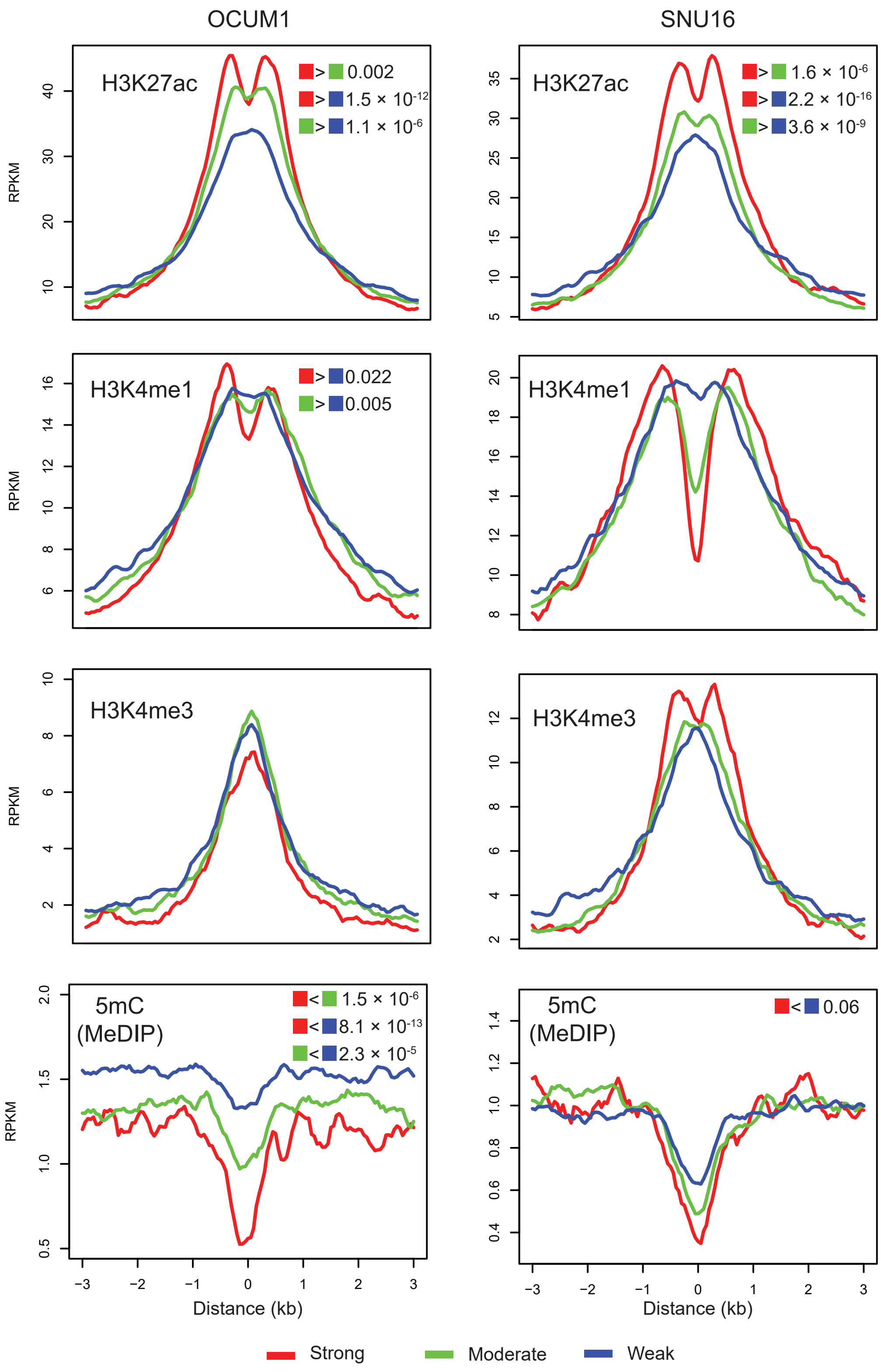

Supplementary Figure 8: Average profiles of H3K27ac, H3K4me1, H3K4me3 ChIP-seq and DNA methylation (5mC) for CapSTARR-seq enriched regions (CaPERs) classified as weak, moderate or strong in OCUM1 (left panels) or in SNU16 (right panels). Statistically significant differences calculated on the regions within 1k bases flanking the summits of CaPERs.

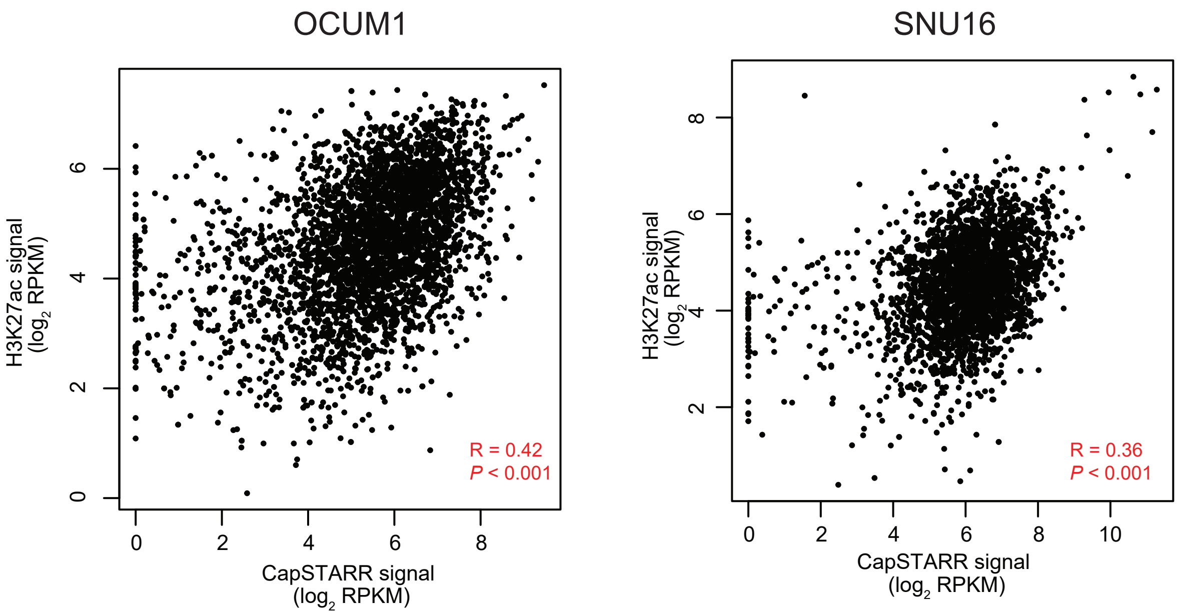

Supplementary Figure 9. Comparison of H3K27ac signals (log2 RPKM) and CapSTARR-seq signals (log2 RPKM) over the H3K27ac active CaPERs in OCUM1 and SNU16 cells. R: Pearson's correlation coefficient. *P*-value: Pearson's correlation test.

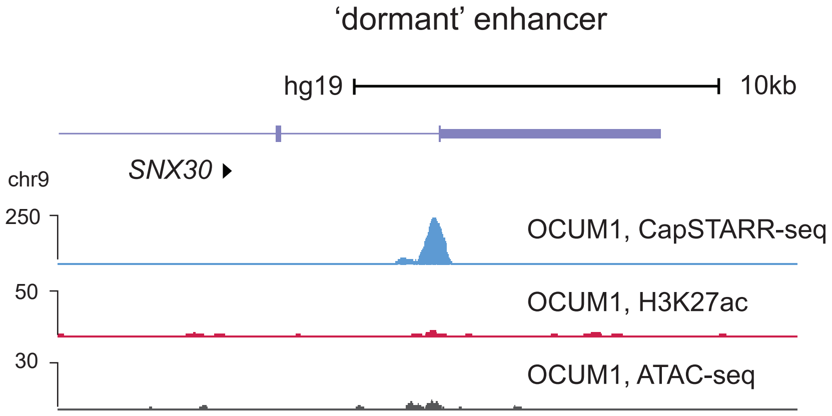

Supplementary Figure 10: CapSTARR-seq (blue), H3K27ac ChIP-seq (red) and ATAC-seq (black) tracks at *SNX30* locus in OCUM1 cells. An OCUM1 'dormant' enhancer exhibits high CapSTARR-seq signals but no H3K27ac and ATAC-seq enrichment.

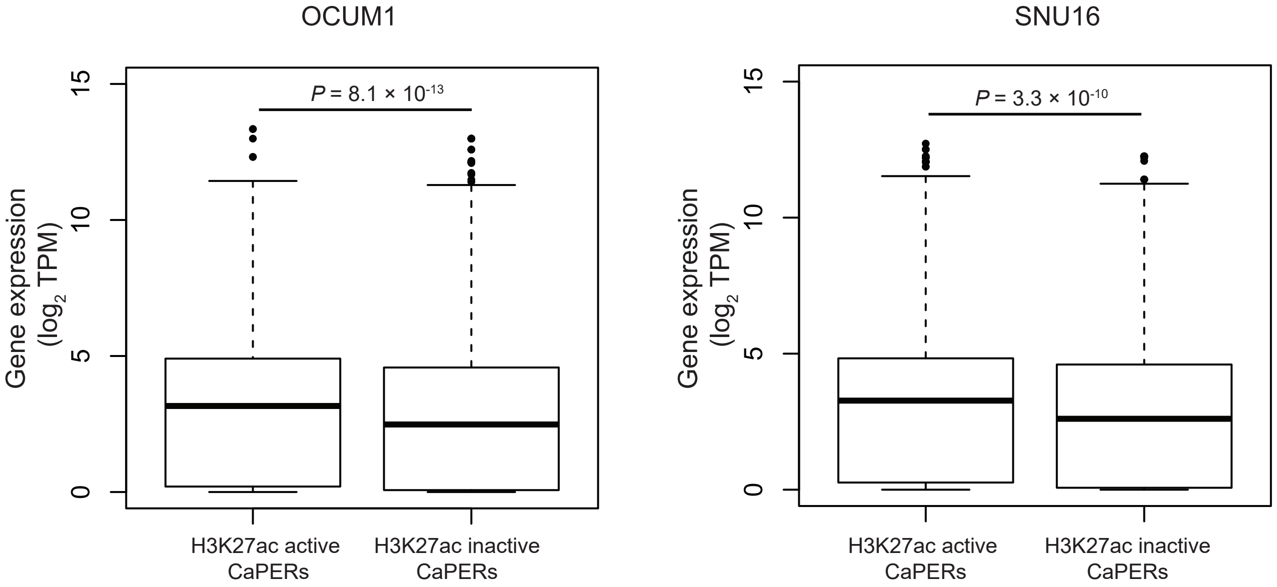

Supplementary Figure 11: Differences in expression levels ($\log_2$ TPM) between genes nearby H3K27ac active and H3K27ac inactive CapSTARR-seq enriched regions (CaPERs) in OCUM1 and SNU16 cells.
P-values: Mann–Whitney U test.

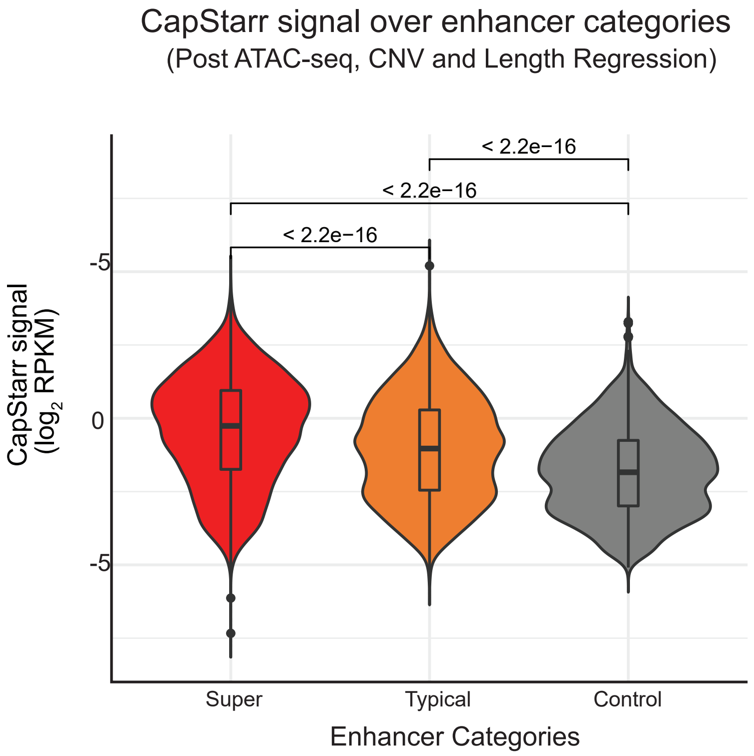

Supplementary Figure 12: Differences in corrected CapSTARR-seq signals (log2 RPKM) over enhancer categories in OCUM1 cells. Effects of DNA accessibility, DNA copy number and region length of predicted enhancers were regressed out from Capstarr-seq signal using a generalized linear model (GLM). *P*-values are calculated using the Mann–Whitney U test.

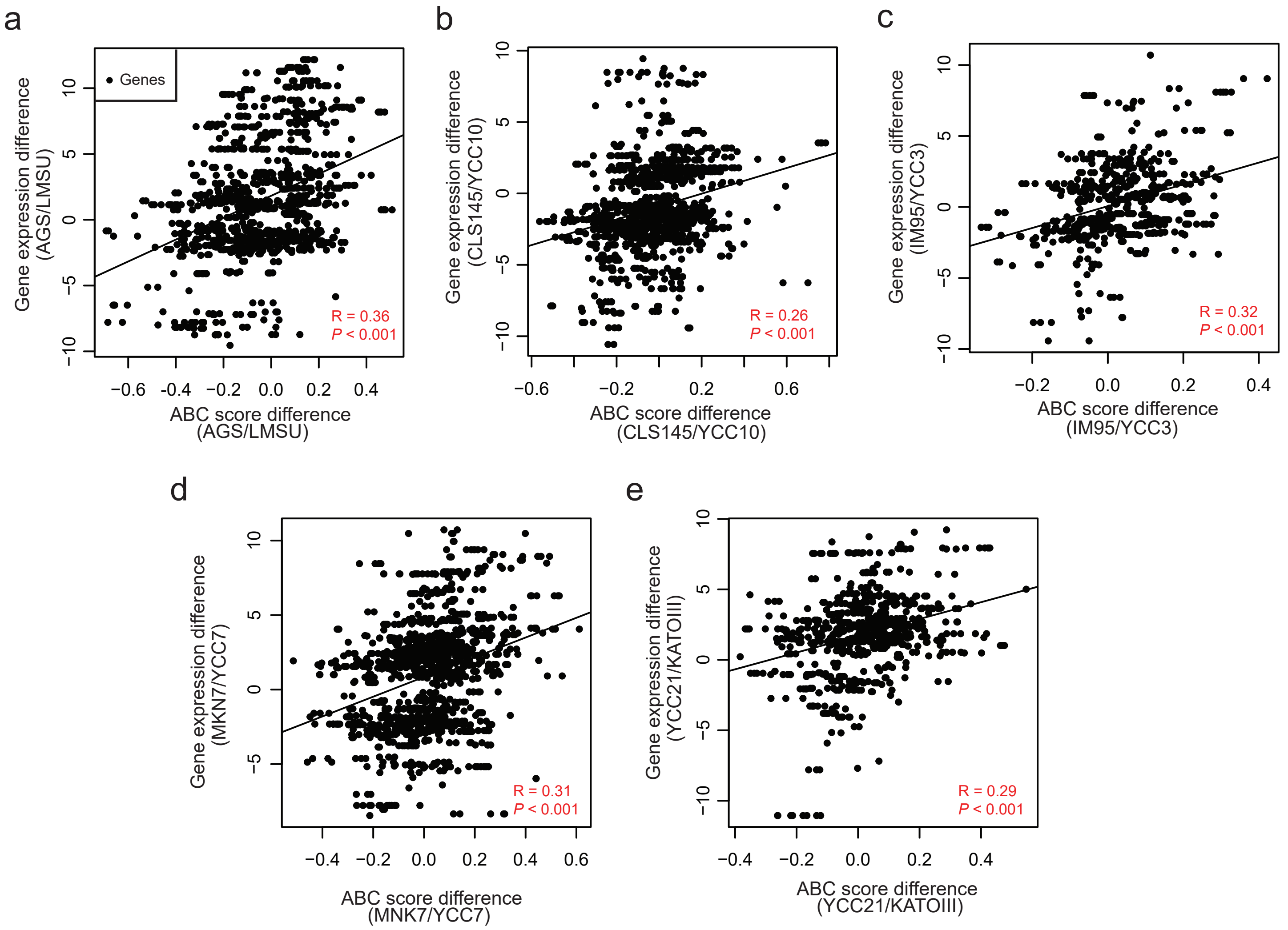

Supplementary Figure 13: Comparison of observed gene expression differences and ABC score differences between **a)** AGS and LMSU, **b)** CLS145 and YCC10, **c)** IM95 and YCC3, **d)** MKN7 and YCC7 and **e)** YCC21 and KATOIII. Each dot represents a differentially expressed gene between the two cells. Activity of an enhancer is estimated as the H3K27ac signal. R: Pearson's correlation coefficient. *P*-value: Pearson's correlation test.

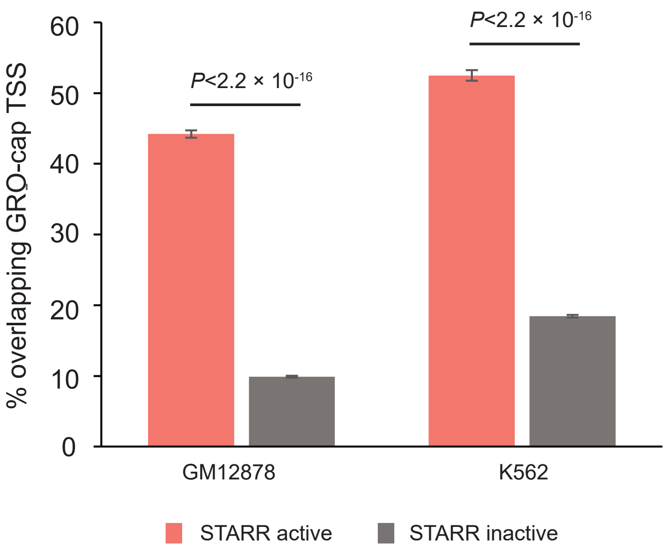

Supplementary Figure 14: The percentage of STARR active and inactive elements within H3K27ac active enhancers overlapping GRO-cap TSS. The error bars indicate the s.e.m. calculated for a sample of binary trials, centered on the observed success rate. *P* values are from a two-sided Fisher's exact test.

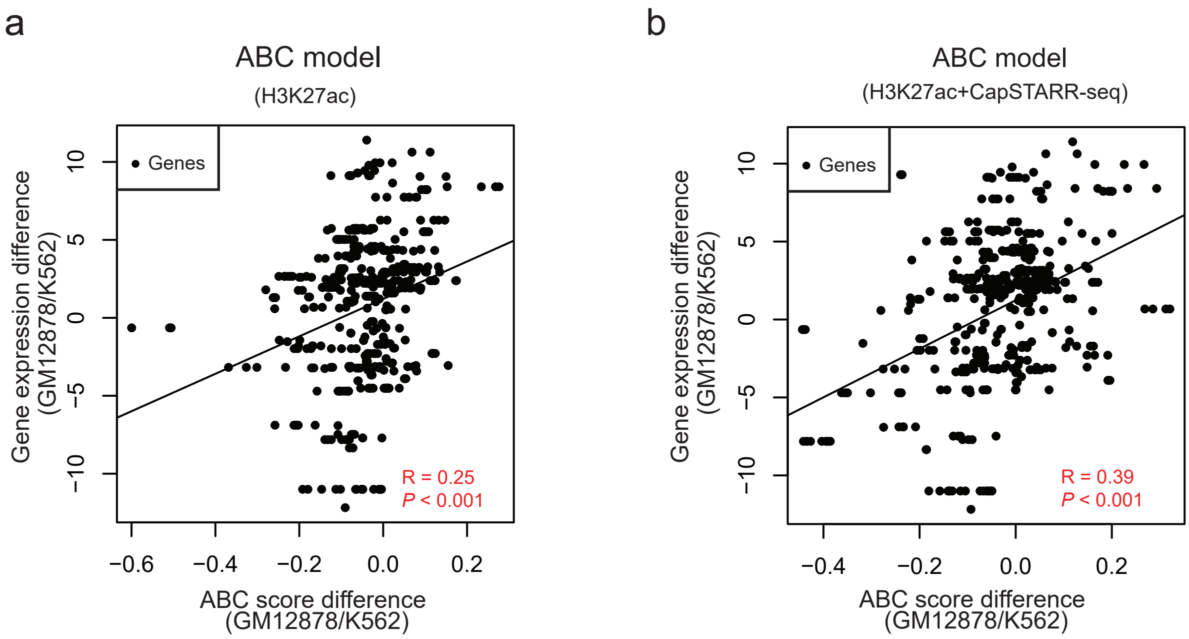

Supplementary Figure 15: Validation of the ABC model. a) Comparison of ABC score differences and observed gene expression differences between GM12878 and K562 cells. Each dot represents a differentially expressed gene between GM12878 and K562 cells. Activity of an enhancer is estimated as the H3K27ac signal. R: Pearson's correlation coefficient. *P*-value: Pearson's correlation test. **b)** Comparison of ABC score differences and observed gene expression differences between GM12878 and K562 cells. Activity of an enhancer is estimated as the geometric mean of H3K27ac-enrichment and CapSTARR-seq signal. R: Pearson's correlation coefficient. *P*-value: Pearson's correlation test.

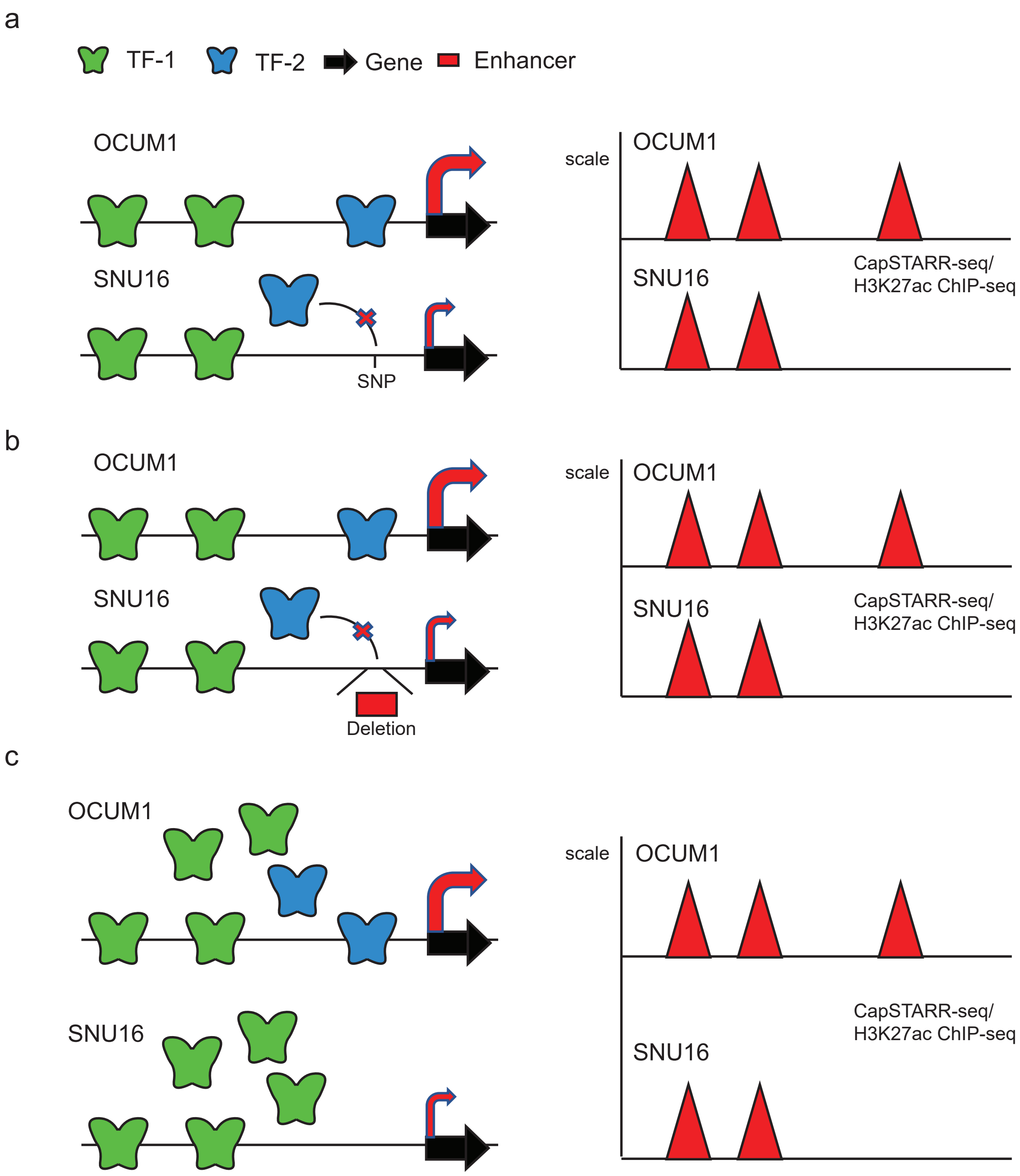

Supplementary Figure 16: Schematic diagram of ‘*cis*-model’ (panel a, b) and ‘*trans*-model’ (panel c).
a) Existence of some SNP causes differential TF binding enrichment, resulting in differential CapSTARR-seq/H3K27ac ChIP-seq signals over the enhancer.
b) Copy number variation of the enhancer region causes differential TF binding enrichment, resulting in differential CapSTARR-seq/H3K27ac ChIP-seq signals over the enhancer.
c) Low expression of some TF results in the disappearance of the CapSTARR-seq/H3K27ac ChIP-seq signal over the enhancer region.

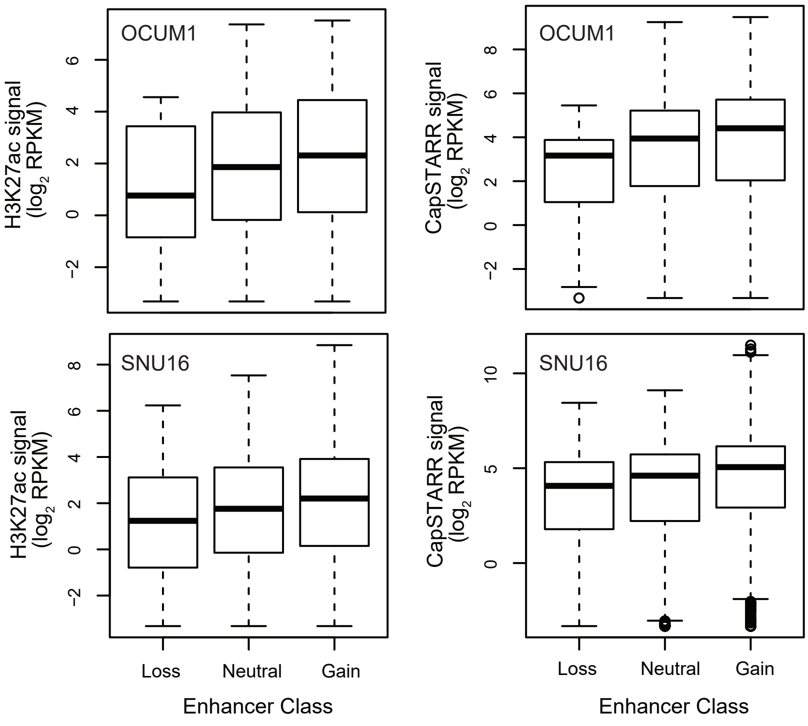

Supplementary Figure 17: Differences in H3K27ac and CapSTARR-seq singals (log₂ RPKM) among three enhancer classes (Loss, Neutral and Gain). Loss enhancers are those with DNA copy number smaller than 2. Neutral enhancers are those with DNA copy number equal to 2. Gain enhancers are those with DNA copy number larger than 2.

ING1 expression~ING1 Copy Number+Enhancer Copy number

	Estimate	Std. Error	t value	Pr(>|t|)
(Intercept)	7.35921	0.10726	68.609	< 2e-16
ING1 Copy Number	0.39068	0.08011	4.877	1.54e-06
Enhancer Copy Number	0.33517	0.09831	3.409	0.000715

Supplementary Figure 18: Results of multiple linear regression model for *ING1* expression of GC samples in the TCGA cohort. Both the copy number of the *ING1* gene itself and the enhancer have a significant effect on *ING1* expression.

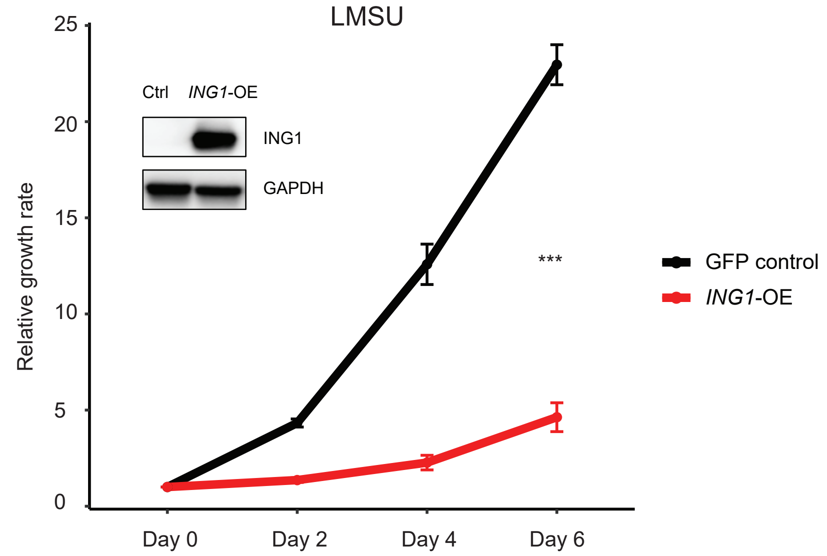

Supplementary Figure 19: Relative cell proliferation rate after *ING1* overexpression in LMSU. *ING1* overexpression led to a decrease in cell proliferation capacity. **P* < 0.05, ***P* < 0.01, and ****P* < 0.001 by 2-sided Student's t-test. Error bars indicate the SD. All data are representative of 3 independent experiments.

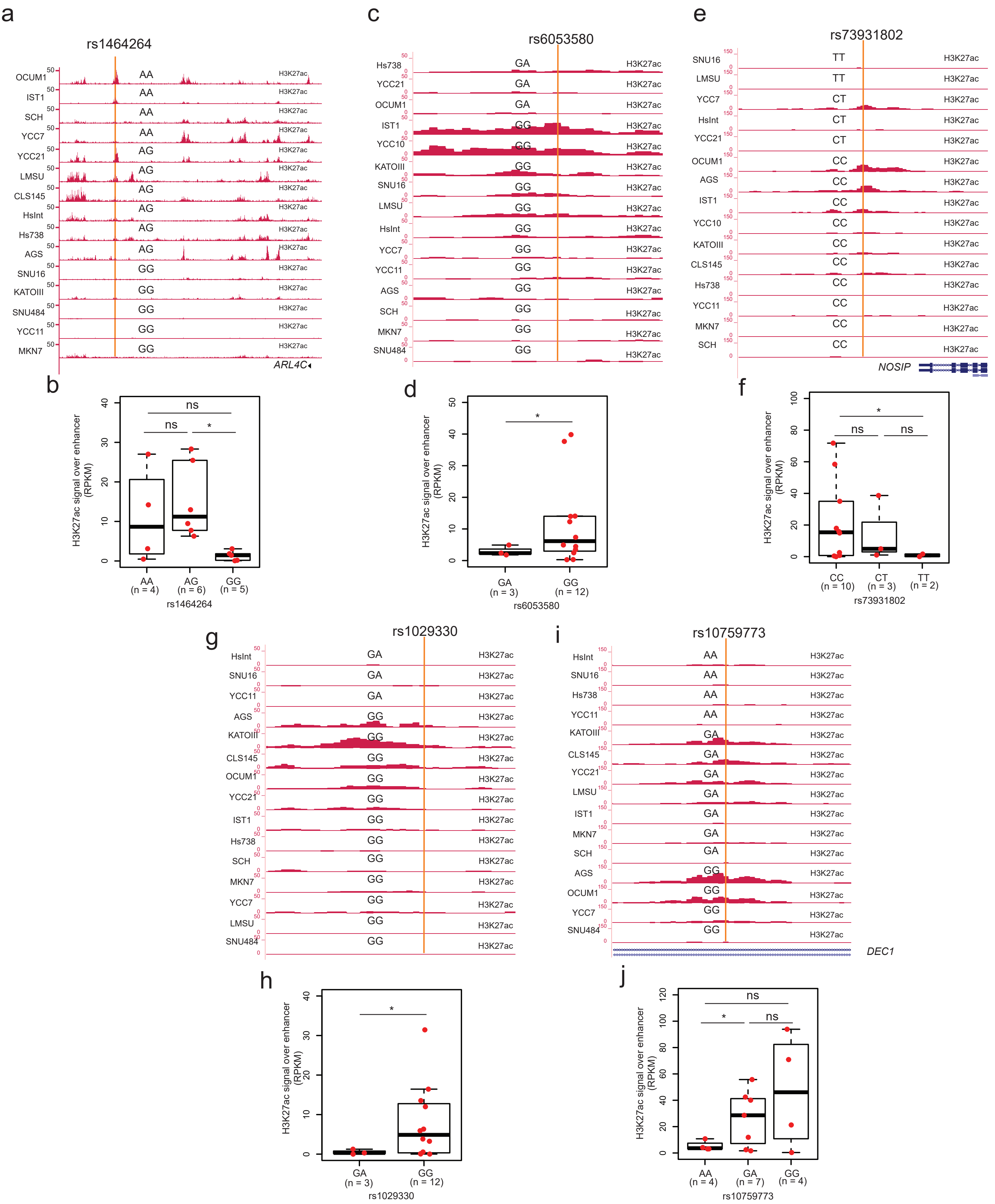

Supplementary Figure 20: Five haQTLs exhibit a significant correlation between histone acetylation levels over the enhancer region and the haQTL genotype. H3K27ac ChIP-seq tracks in GC lines are shown at haQTL **a)** rs1464264 ,**c)** rs6053580, **e)** rs73931802, **g)** rs1029330, and **i)** rs10759773. The haQTL genotype is annotated above the corresponding H3K27ac ChIP-seq track in each cell line. The boxplots show the difference in H3K27ac signals (RPKM) over the enhancer region in different groups of GC lines with different SNP **b)** rs1464264 ,**d)** rs6053580, **f)** rs73931802, **h)** rs1029330, and **j)** rs10759773 genotypes. *: *P* < 0.05, ns: not significant, Student's t-test.

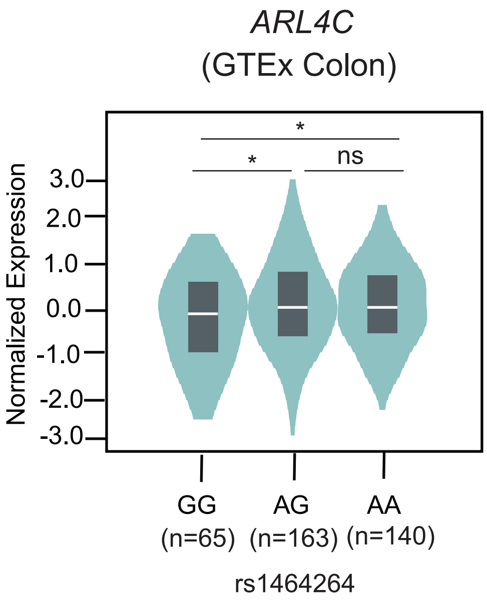

Supplementary Figure 21: Differences in expression of *ARL4C* in three groups of colon samples with different SNP rs1464264 genotypes (GG, AG and AA) collected by the Genotype-Tissue Expression (GTEx) project (n= 368).
*: *P* < 0.05, ns: not significant, Student's t-test.

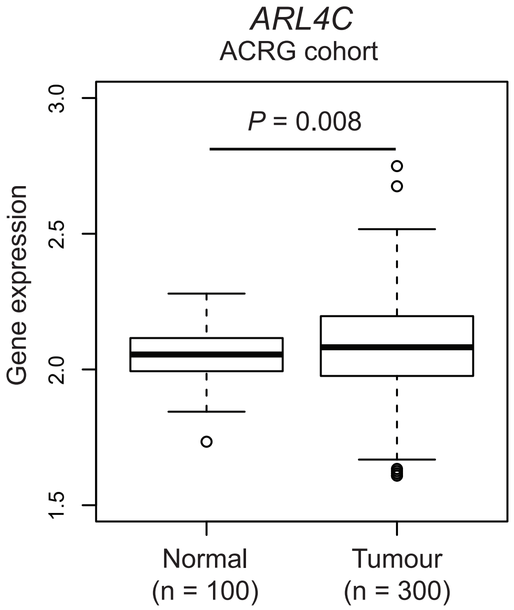

Supplementary Figure 22: Expression of *ARL4C* in normal gastric (n=100) and GC samples (n=300) from the ACRG cohort. *P*-value: Student's t-test.

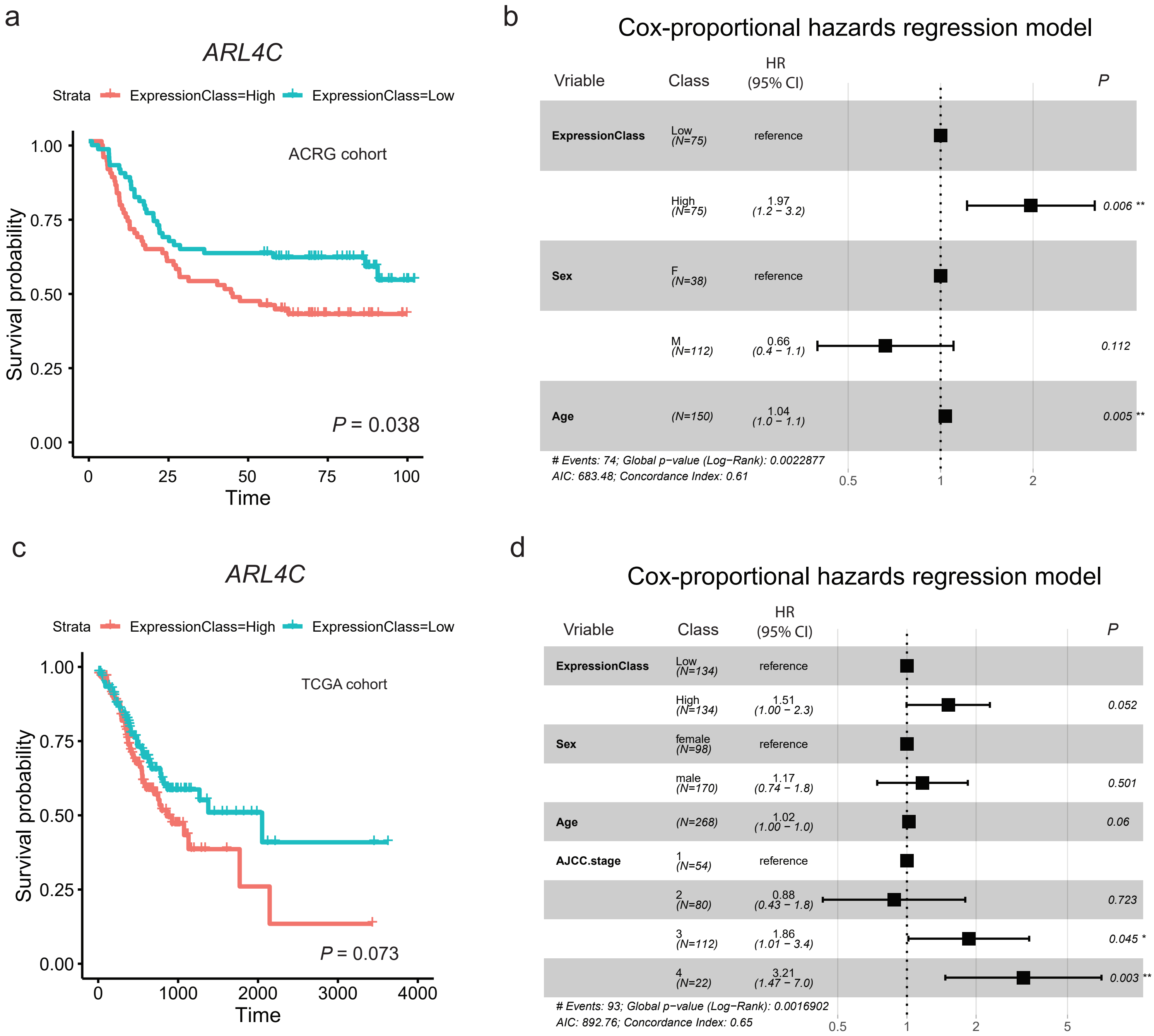

Supplementary Figure 23. Survival analysis for *ARL4C* in the ACRG and TCGA cohort. Kaplan–Meier survival curve comparing patient groups with samples exhibiting low (green) and high (red) expression of *ARL4C* in **a)** the ACRG cohort and **c)** the TCGA cohort. *P*-value is calculated using the Log-rank test. Results of multivariate analysis by Cox-proportional hazards regression model for overall survival in **b)** the ACRG cohort and **d)** the TCGA cohort. CI, confidence interval; HR, hazard ratio.

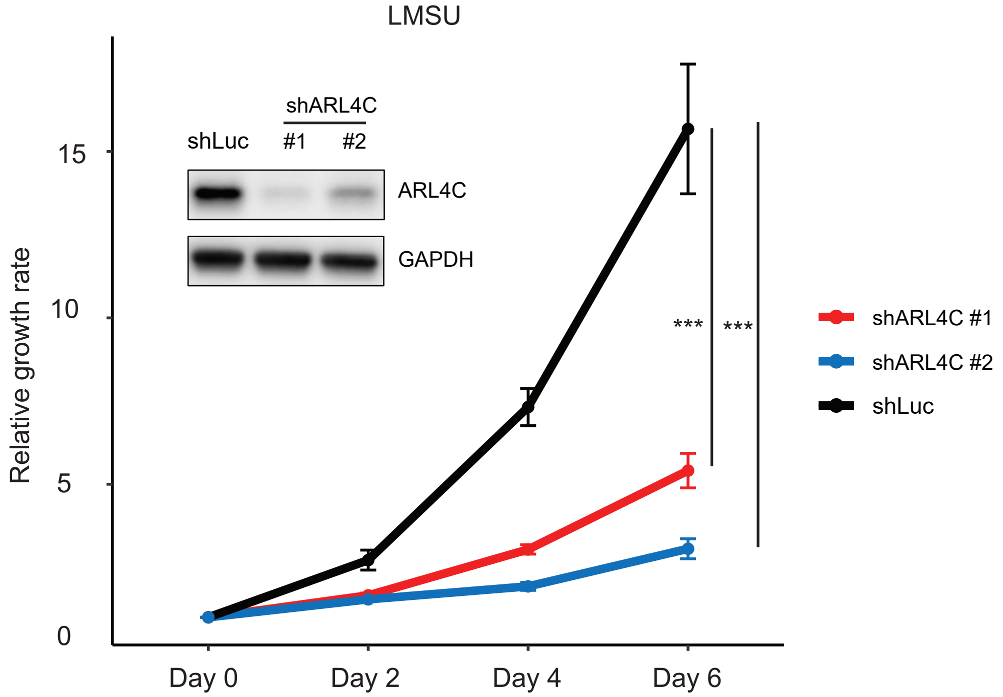

Supplementary Figure 24: Relative cell proliferation rate after *ARL4C* knockdown in LMSU. *ARL4C* knockdown led to a decrease in cell proliferation capacity. *$P < 0.05$, **$P < 0.01$, and ***$P < 0.001$ by 2-sided Student's t-test. Error bars indicate the SD. All data are representative of 3 independent experiments.

a

Transcription factor	Motif	*P*-value	% Target regions with motif
Fra1	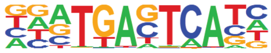	1×10^{-166}	32.08%
Fra2	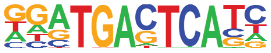	1×10^{-163}	29.97%
Atf3	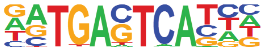	1×10^{-155}	34.01%
BATF	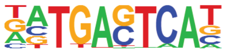	1×10^{-154}	33.62%
JunB	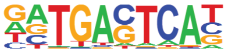	1×10^{-153}	31.03%

b

Transcription factor	Motif	*P*-value	% Target regions with motif
Fosl2	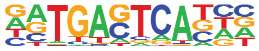	1×10^{-79}	9.93%
BATF	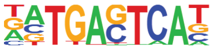	1×10^{-78}	15.57%
Jun-AP1	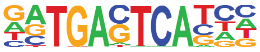	1×10^{-77}	8.16%
Fra2	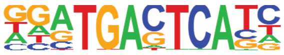	1×10^{-76}	12.56%
Fra1	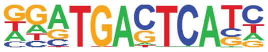	1×10^{-75}	13.83%
Atf3	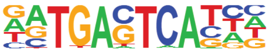	1×10^{-69}	15.15%

Supplementary Figure 25: Top 5 transcription factor binding enrichments at OCUM1-specific enhancers defined based on a) CapSTARR and H3K27a paterns or b) only H3K27ac as determined by using HOMER *de novo* motif analysis.

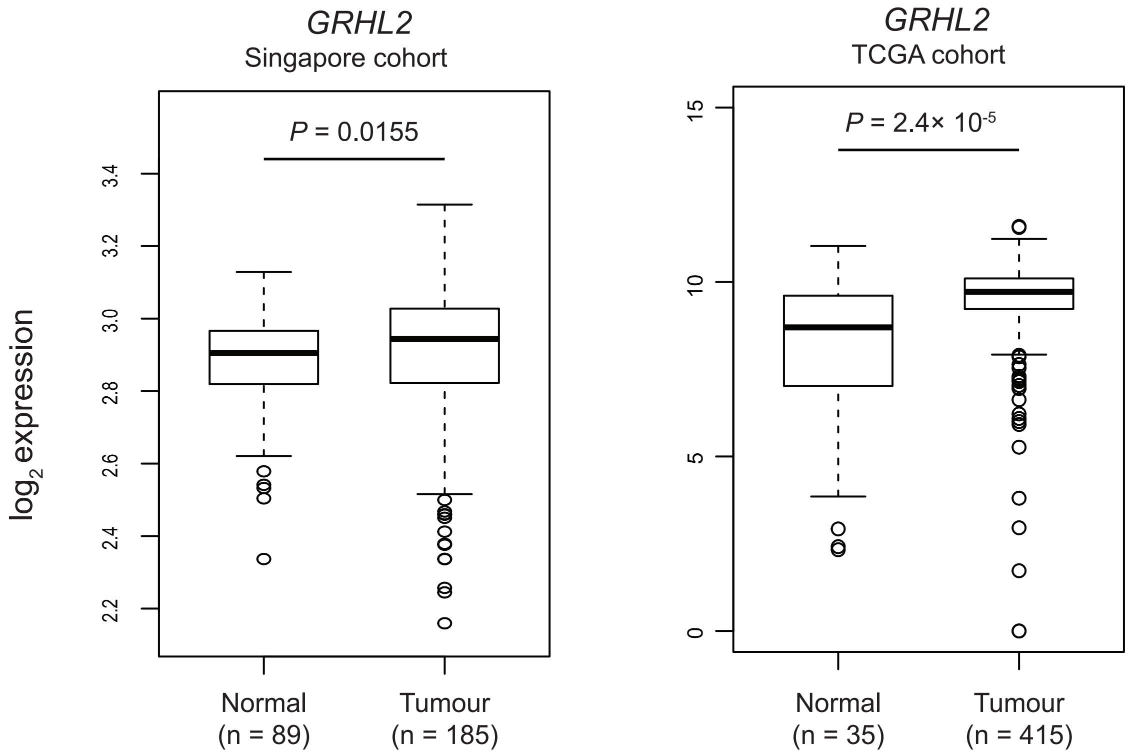

Supplementary Figure 26: Expression of *GRHL2* in normal gastric and GC samples from the Singapore cohort (Left) or the TCGA cohort (Right). *P*-value: Mann–Whitney U test.
